# Blood pressure variability supersedes heart rate variability as a real-world measure of dementia risk

**DOI:** 10.1038/s41598-024-52406-8

**Published:** 2024-01-22

**Authors:** Joseph E. Ebinger, Matthew P. Driver, Tzu Yu Huang, Jose Magraner, Patrick G. Botting, Minhao Wang, Peng-Sheng Chen, Natalie A. Bello, David Ouyang, John Theurer, Susan Cheng, Zaldy S. Tan

**Affiliations:** 1 Department of Cardiology, Smidt Heart Institute, Cedars-Sinai Medical Center, Los Angeles, CA USA; 2 Departments of Neurology and Medicine, Cedars-Sinai Medical Center, Los Angeles, CA USA; 3 Department of Medicine, David Geffen School of Medicine, University of California Los Angeles, Los Angeles, CA USA

**Keywords:** Cardiology, Medical research

## Abstract

Blood pressure variability (BPV) and heart rate variability (HRV) have been associated with Alzheimer’s Disease and Related Dementias (ADRD) in rigorously controlled studies. However, the extent to which BPV and HRV may offer predictive information in real-world, routine clinical care is unclear. In a retrospective cohort study of 48,204 adults (age 54.9 ± 17.5 years, 60% female) receiving continuous care at a single center, we derived BPV and HRV from routinely collected clinical data. We use multivariable Cox models to evaluate the association of BPV and HRV, separately and in combination, with incident ADRD. Over a median 3 [2.4, 3.0] years, there were 443 cases of new-onset ADRD. We found that clinically derived measures of BPV, but not HRV, were consistently associated with incident ADRD. In combined analyses, only patients in both the highest quartile of BPV and lowest quartile of HRV had increased ADRD risk (HR 2.34, 95% CI 1.44–3.81). These results indicate that clinically derived BPV, rather than HRV, offers a consistent and readily available metric for ADRD risk assessment in a real-world patient care setting. Thus, implementation of BPV as a widely accessible tool could allow clinical providers to efficiently identify patients most likely to benefit from comprehensive ADRD screening.

## Introduction

Cardiovascular measures of autonomic dysfunction have been associated with Alzheimer’s Disease and Related Dementias (ADRD) in rigorously controlled studies and proposed as a method for identifying patients at risk for or experiencing subclinical cognitive dysfunction ^1–4^. Blood pressure variability (BPV) represents the most well studied of these factors, with higher BPV associated with incident ADRD in numerous prospective cohort studies ^5–7^. Importantly, obtaining BPV data requires repeated blood pressure measurements, with most studies spanning years. Further, blood pressure values used to determine BPV in these studies were captured using high-fidelity protocols that are not often used in clinical practice ^8–11^. Therefore, while promising, it remains unclear if BPV derived from clinically generated blood pressure data may offer similar or even any valuable information with respect to ADRD risk stratification in a real-world clinical care setting.

Heart rate variability (HRV) represents an alternative measure of autonomic tone that has been associated with cognitive dysfunction and which can be obtained over a much shorter period of time ^12,13^. Analysis of EKGs from the Multi-Ethnic Study of Atherosclerosis (MESA) Study found an association between higher HRV and better cognitive performance across multiple domains ^14^. Compared with BP assessment, EKG ascertainment is less susceptible to measurement error, potentially improving the ability of this modality to be utilized in clinical practice. Conversely, EKGs are not performed as frequently as blood pressure measurements, potentially limiting clinical utility.

There exists an urgent need to develop efficient and cost-effective approaches to identifying patients at high risk for developing ADRD, particularly earlier in the course of disease progression ^15,16^, given ongoing aging of the population ^17,18^ and amid concerns that existing approaches to disease screening are considered relatively inaccurate and burdensome ^19–21^. The rapid uptake of electronic health records and digital EKG capture has created an abundance of longitudinal blood pressure and heart rate measures that are typically collected and stored for individual patients as they age through their care within a healthcare system. As such, BPV and HRV represent potentially readily accessible clinical tools for identifying and prioritizing patients who could benefit from more comprehensive ADRD screening protocols, however, given the recognized limitations of each modality, which method maintains optimal predictive capacity in a real-world clinical setting remains unknown. To address this knowledge gap, we evaluated the association of BPV and HRV with subsequent diagnosis of ADRD, using clinical data longitudinally collected from a diverse cohort of patients cared for in a large urban multi-site health system.

## Results

A total of 631,216 patients had at least 1 outpatient encounter at which a BP was recorded during the clinical assessment period, of whom 51,147 had an outpatient visit every calendar year from 2013 through 2016. Following exclusion of 2270 individuals under age 18, 335 with a history of ADRD, 334 who died during the final year of the clinical assessment period, and 4 with non-physiologic BP measurements, there was a total of 48,204 patients receiving consistent care during the clinical assessment period. Of these 7270 (15.1%) had at least 1 qualifying EKG (Supplemental Fig. 1). The median follow-up time for both the full and EKG cohorts was 3.0 [2.4, 3.0] years, during which time 443 new dementia cases occurred.

The average age of the BP cohort was 54.9 ± 17.5 years, of whom 29,011 (60.2%) were female. The most common comorbid condition was diabetes mellitus (11.9%) followed by coronary artery disease (11.3%), heart failure (8.6%), and kidney disease (7.4%). On average, patients had 15.4 ± 13.9 blood pressure measurements during the clinical assessment period, with a mean SBP of 124.0 ± 12.1 mmHg and DBP of 73.8 ± 7.2 mmHg, with 13,539 (28.1%) prescribed at least 1 antihypertensive medication. By comparison, the average age of the EKG Cohort was 68.1 ± 15.8 years of age, with 4015 (55.2%) females. Coronary artery disease was the most common comorbid condition (27.0%), followed by heart failure (23.5%), diabetes mellitus (19.4%), and atrial fibrillation/flutter (17.3%). Participants had on average 23.9 ± 18.4 BP measurements, with a mean SBP of 127.0 ± 11.8 mmHg and DBP of 73.0 ± 7.4 mmHg, with 3351 (46.1%) prescribed at least 1 antihypertensive medication. The median number of qualifying EKGs was 2.0 [1.0, 3.0], with an average heart rate of 73.9 ± 24.2 beats per minute (Table 1). By comparison, the BP and EKG cohorts were generally older, more frequently non-Hispanic White, and had more BP recordings in the EHR than patients not meeting inclusion criteria (Table 1). Frequency of BP measurements per patient can be found in Supplemental Table 1.

**Table 1:** Baseline characteristics of blood pressure and EKG cohorts, as well as all ambulatory care patients prior to eligibility screening.

Characteristic	All ambulatory care patients (n = 631,216)	BP cohort (n = 48,204)	EKG cohort (n = 7270)
*Demographic characteristics*			
Age, years, mean (SD)	46.26 (20.65)	54.87 (17.50)	68.11 (15.77)
Age, ≥ 65 years, n (%)	140,988 (22.3)	15,397 (31.9)	4517 (62.1)
Female, n (%)	358,869 (56.9)	29,011 (60.2)	4015 (55.2)
Race/ethnicity, n (%)			
Asian	51,134 (8.1)	4437 (9.2)	483 (6.6)
Hispanic/Latinx	75,291 (11.9)	5322 (11.0)	691 (9.5)
Non-Hispanic Black	59,163 (9.4)	6385 (13.2)	1111 (15.3)
Non-Hispanic White	357,461 (56.6)	29,483 (61.2)	4735 (65.1)
Other^a^	28,359 (4.5)	1423 (3.0)	214 (2.9)
Smoking status, n (%)			
Current	39,683 (6.3)	2266 (4.7)	320 (4.4)
Former	119,872 (19.0)	14,011 (29.1)	2832 (39.0)
Never	410,407 (65.0)	31,927 (66.2)	4118 (56.6)
*Clinical characteristics*			
Follow-up length, mean (SD), years	–	2.45 (0.97)	2.37 (1.03)
Follow-up length, median [IQR], years	–	3.00 [2.36, 3.00]	3.0 (2.0, 3.0)
Number of blood pressures recorded during study period, mean (SD)	6.88 (11.17)	15.39 (13.86)	23.86 (18.38)
Number of blood pressures recorded during study period, median [IQR]	3.00 [1.00, 8.00]	12.00 [8.00, 18.00]	19.0 [12.0, 30.0]
Diabetes mellitus, n (%)	–	5721 (11.9)	1409 (19.4)
Renal disease, n (%)	–	3559 (7.4)	1250 (17.2)
Atrial fibrillation or atrial flutter, n (%)	–	3376 (7.0)	1261 (17.3)
Coronary artery disease, n (%)	–	5441 (11.3)	1960 (27.0)
Metastatic malignancy, n (%)	–	872 (1.8)	318 (4.4)
Myocardial Infarction, n (%)	–	1807 (3.7)	814 (11.2)
Heart failure, n (%)	–	4122 (8.6)	1709 (23.5)
Stroke, n (%)	–	2543 (5.3)	851 (11.7)
*Blood pressure characteristics*			
Antihypertensive use, n (%)	–	13,539 (28.1)	3351 (46.1)
Mean systolic blood pressure, mean (SD), mmHg	122.69 (15.16)	123.96 (12.09)	126.99 (11.78)
Mean systolic blood pressure, median [IQR], mmHg	121.67 [112.00, 132.00]	123.43 [115.30, 131.80]	127.0 [119.0, 134.0]
Mean diastolic blood pressure, mean (SD), mmHg	74.03 (9.36)	73.77 (7.18)	72.97 (7.43)
Mean diastolic blood pressure, median [IQR], mmHg	74.00 [68.00, 80.00]	73.46 [68.81, 78.40]	73.0 [68.0, 78.0]
*Heart rate characteristics*			
Number of EKGs, mean (SD)	–	–	2.74 (3.59)
Number of EKGs, median [IQR]	–	–	2.0 [1.0, 3.0]
Heart rate, mean (SD)	–	–	73.91 (24.18)
Heart rate, median [IQR]	–	–	70.0 [62.0, 81.0]
Number of QRS complexes, mean (SD)	–	–	10.76 (2.7)
Number of QRS complexes, median [IQR]	–	–	10 [9, 12]

^a^Other race includes American Indian/Alaska Native, Native Hawaiian or other Pacific Islander, and Other.

*SD* standard deviation, *IQR* interquartile range.

**Figure 1:**
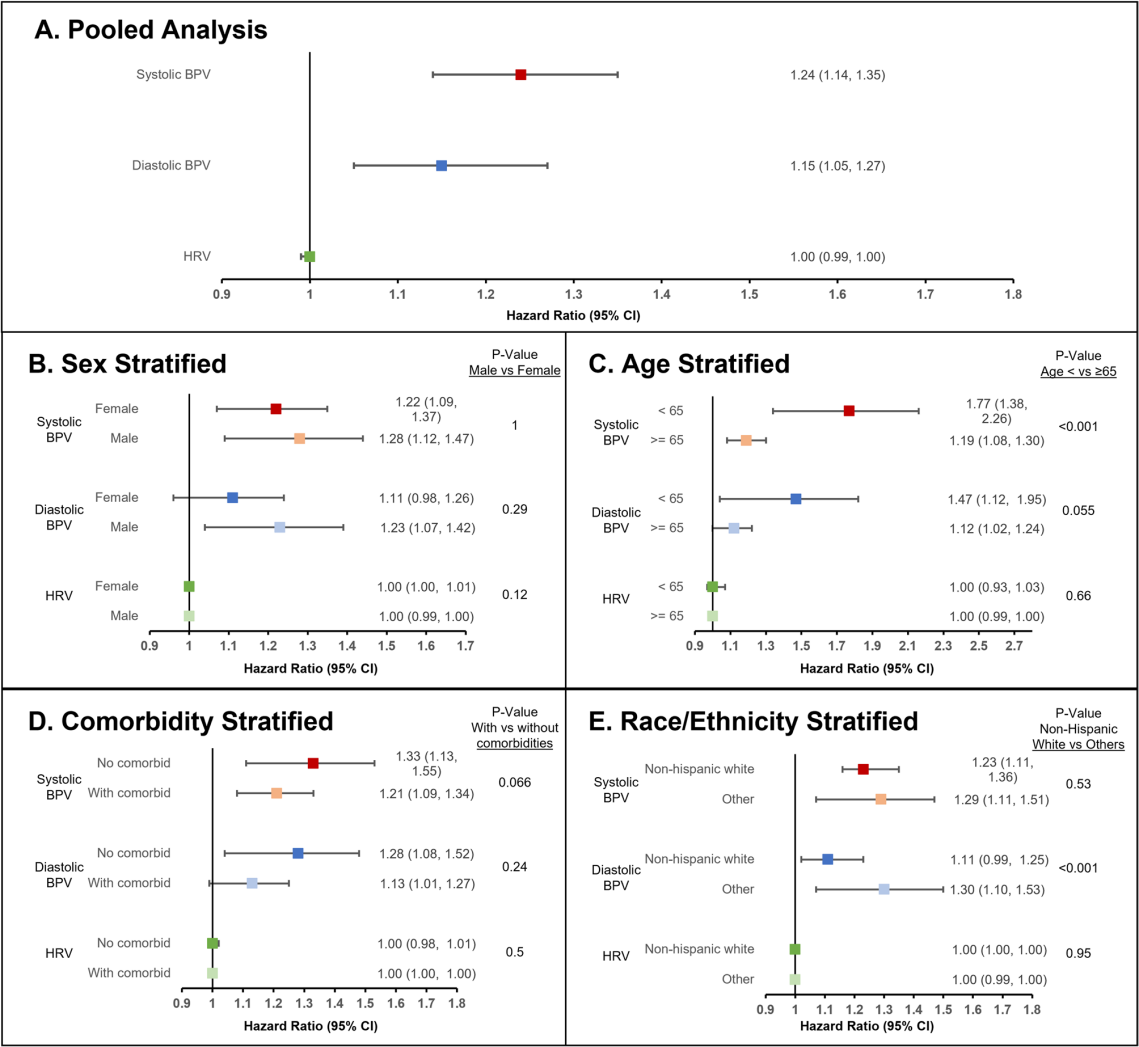
Association of blood pressure and heart rate variability with incident Alzheimer’s Disease and Related Dementias in the EKG cohort. Incident dementia risk associated with systolic BPV, diastolic BPV, and HRV (**A**) in the pooled cohort, and in stratified analyses by (**B**) sex, (**C**) age, (**D**) number of comorbidities (0 vs. ≥ 1), and (**E**) race/ethnicity (Non-Hispanic White vs. Others). Cox models adjusted for age, sex, race/ethnicity, number of visits, use of antihypertensive medications, smoking status, diabetes mellitus, chronic kidney disease, atrial fibrillation/atrial flutter, coronary artery disease, mean systolic and diastolic blood pressure, presence of any metastatic malignancy, myocardial infarction, heart failure, stroke, number of EKGs, and heart rate. Patients censored at last follow up visit or death prior to the end of the study period, whichever occurred later. Analyses exclude patients of unknown race due to model convergence. Abbreviations: *BPV* blood pressure variability, *CI* confidence interval, *HRV* heart rate variability.

In multivariable Cox proportional hazards models, the risk of incident ADRD increased as BPV increased for both systolic (Hazard Ratio 1.24, 95% CI 1.14–1.35) and diastolic (1.15, 1.05–1.27) VIM in the BP cohort, while no association between ADRD and HRV was appreciated in the EKG cohort (1.00, 0.99–1.00) (Fig. 1A). Increasing age, more clinic visits, diabetes mellitus, prior myocardial infarction, and heart failure were all associated with increased risk of ADRD (Supplemental Table 2). Of note, mean systolic and diastolic BP were not associated with ADRD. Results were similar when BPV and HRV were included in the same model using the EKG cohort (Supplemental Table 3). Similar findings were noted following stratification by age and sex without significant risk difference between sexes or age strata (Fig. 1B,C). Similarly, BPV, particularly systolic VIM, remained significantly associated with incident ADRD following stratification by number of comorbid conditions, and race (Fig. 1D,E). There were generally not significant differences between groups, except for systolic BPV following age stratification (P-value for interaction < 0.001) and diastolic BPV following race stratification (P-value for interaction < 0.001). The c-statistics for the primary models can be found in Supplemental Table 4. Survival analysis demonstrated a significantly lower disease-free survival among patients with a systolic VIM above the median for the BP cohort (Supplemental Fig. 2). In secondary analyses, we repeated the BPV models using only the smaller EKG cohort with similar to slightly higher hazard ratios for both systolic (1.37, 1.16–1.61) and diastolic BPV (1.15, 1.05–1.27). Similar findings were again noted following stratification by age and sex, except for individuals < 65 years, for whom there were too few events for analysis (7 ADRD cases out of 2753, 0.25%) (Table 2).

**Table 2:** Association of combined systolic blood pressure and heart rate variability with incident Alzheimer’s Disease and Related Dementias in the EKG cohort, overall and stratified by sex and age.

Outcome	Overall (n = 7270)		Sex stratified			Age stratified	
			Female (n = 4015)	Male (n = 3255)	p-value^b^	Age ≥ 65 (n = 4517)	Age < 65 (n = 2753)^c^
	Crude HR (95% CI)	Adjusted HR (95% CI)^a^	Adjusted HR (95% CI)^a^	Adjusted HR (95% CI)^a^		Adjusted HR (95% CI)^a^	Adjusted HR (95% CI)^a^
Systolic VIM	**1.56 (1.36, 1.79)**	**1.37 (1.16, 1.61)**	**1.35 (1.08, 1.70)**	**1.41 (1.11, 1.79)**	0.84	**1.38 (1.16, 1.63)**	–
Diastolic VIM	**1.26 (1.09, 1.47)**	1.18 (0.99, 1.40)	1.09 (0.85, 1.38)	**1.34 (1.05, 1.71)**	0.14	1.18 (0.99, 1.41)	–

*CI* confidence interval, *HR* hazard ratio, *VIM* variation independent of the mean.

^a^Cox models adjusted for age, sex, race/ethnicity, number of visits, use of antihypertensive medications, smoking status, diabetes mellitus, chronic kidney disease, atrial fibrillation/atrial flutter, coronary artery disease, mean systolic and diastolic blood pressure, presence of any metastatic malignancy, myocardial infarction, heart failure, stroke, number of EKGs, and heart rate. Patients censored at last follow up visit or death prior to the end of the study period, whichever occurred later. Analyses exclude patients of unknown race due to model convergence.

^b^P-values for sex interaction, i.e. difference in adjusted HRs between males and females.

^c^Too few events (n = 7) for modeling.

Significant values are in bold.

**Table 3:** Association of combined systolic blood pressure and heart rate variability with incident Alzheimer’s Disease and Related Dementias in the EKG cohort using all available EKGs, overall and stratified by sex.

Outcome	Overall (n = 7270)		Sex stratified		p-value^b^
			Female (n = 4015)	Male (n = 3255)	
	Crude HR (95% CI)	Adjusted HR (95% CI)^a^	Adjusted HR (95% CI)^a^	Adjusted HR (95% CI)^a^	
Systolic VIM + HRV					
Low VIM, high HRV (n = 4165)	**Ref**	**Ref**	**Ref**	**Ref**	–
Low VIM, low HRV (n = 1287)	0.95 (0.58, 1.56)	0.98 (0.59, 1.63)	0.65 (0.29, 1.50)	1.34 (0.69, 2.62)	0.188
High VIM, high HRV (n = 1285)	1.94 (1.31, 2.86)	1.32 (0.88, 1.97)	1.17 (0.68, 2.03)	1.58 (0.86, 2.91)	0.482
High VIM, low HRV (n = 533)	3.03 (1.91, 4.82)	**2.34 (1.44, 3.81)**	1.61 (0.80, 3.23)	**3.21 (1.60, 6.46)**	0.176
Diastolic VIM + HRV					
Low VIM, high HRV (n = 4155)	**Ref**	**Ref**	**Ref**	**Ref**	–
Low VIM, low HRV (n = 1297)	1.53 (1.03, 2.28)	1.54 (1.01, 2.33)	1.02 (0.53, 1.96)	2.09 (1.19, 3.68)	0.103
High VIM, high HRV (n = 1295)	1.39 (0.92, 2.09)	1.22 (0.80, 1.86)	1.00 (0.56, 1.76)	1.59 (0.84, 3.00)	0.288
High VIM, low HRV (n = 523)	0.93 (0.45, 1.93)	0.99 (0.47, 2.09)	0.91 (0.35, 2.38)	0.94 (0.28, 3.12)	0.979

*BPV* blood pressure variability, *CI* confidence interval, *HR* hazard ratio, *HRV* heart rate variability, *VIM* variation independent of the mean.

^a^Cox models adjusted for age, sex, race/ethnicity, number of visits, use of antihypertensive medications, smoking status, diabetes mellitus, chronic kidney disease, atrial fibrillation/atrial flutter, coronary artery disease, mean systolic and diastolic blood pressure, presence of any metastatic malignancy, number of EKGs, myocardial infarction, heart failure, stroke, and heart rate. Patients censored at last follow up visit or death prior to the end of the study period, whichever occurred later. Analyses exclude patients of unknown race due to model convergence.

^b^P-values for sex interaction, i.e. difference in adjusted HRs between males and females.

Significant values are in bold.

**Figure 2:**
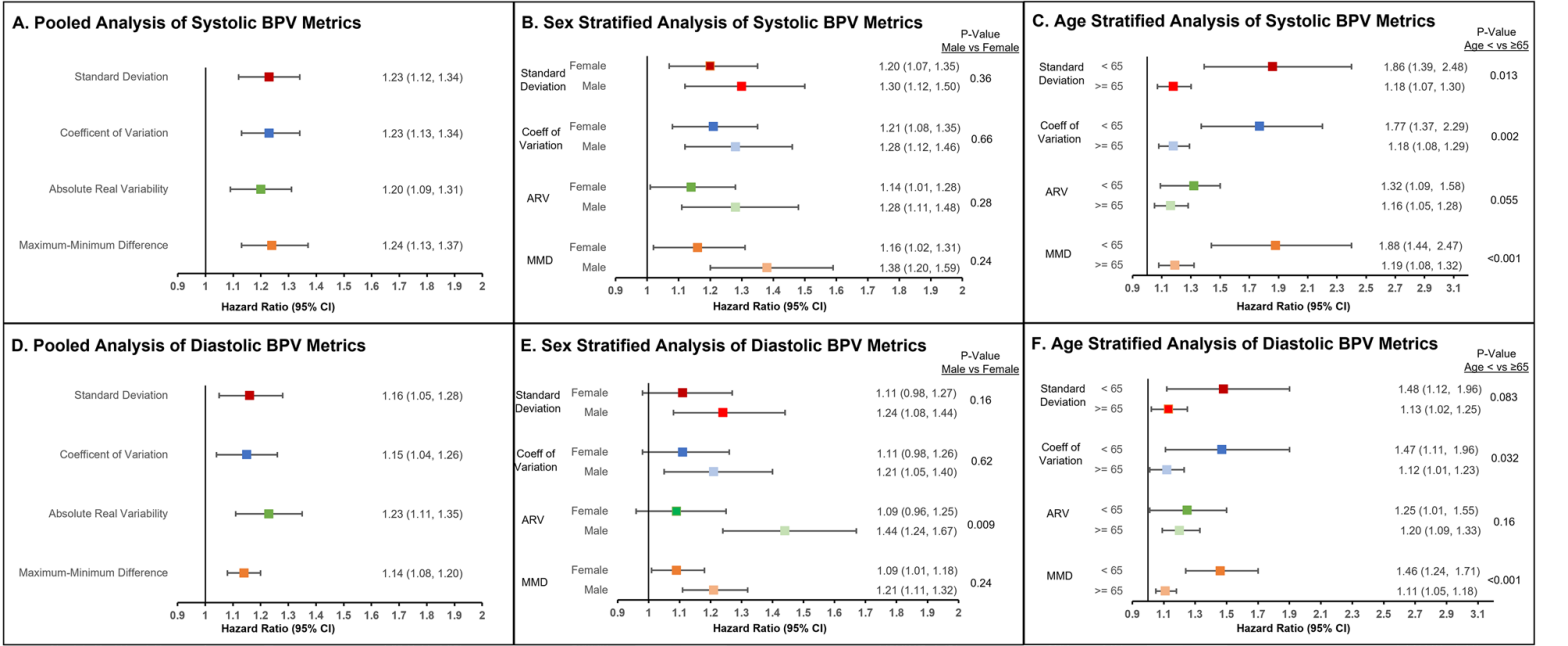
Association of multiple blood pressure variability metrics with incident Alzheimer’s Disease and Related Dementias. Incident dementia risk associated with systolic measures of BPV (A) in the pooled cohort, (**B**) stratified by sex, and (C) stratified by age, as well as diastolic measures of BPV with the same stratification (**D**–**F**). Cox models adjusted for age, sex, race/ethnicity, number of visits, use of antihypertensive medications, smoking status, diabetes mellitus, chronic kidney disease, atrial fibrillation/atrial flutter, coronary artery disease, mean systolic and diastolic blood pressure, presence of any metastatic malignancy, myocardial infarction, heart failure, stroke, number of EKGs, and heart rate. Patients censored at last follow up visit or death prior to the end of the study period, whichever occurred later. Analyses exclude patients of unknown race due to model convergence. Abbreviations: *ARV* absolute real variability, *CI* confidence interval, *MMD* maximum–minimum difference, *SD* standard deviation.

Following categorization based on quartile of systolic BPV and HRV, a total of 4165 (57.3%) patients were categorized as low BPV/high HRV (reference group with presumed lowest risk hemodynamic profile), 1287 (17.7%) as low BPV/low HRV, 1285 (17.7%) as high BPV/high HRV, and 533 (7.3%) as high BPV/low HRV (presumed highest risk hemodynamic profile). In multivariable adjusted Cox models, an association between BPV/HRV category during the clinical assessment period and incident ADRD during the outcome surveillance period was only appreciated among those with high BPV and low HRV (2.34, 1.44–3.81) (Table 3). Similar findings were appreciated when quartiles of BPV and HRV were examined independently (Supplemental Table 5A,B). In sex stratified analyses, the high BPV/low HRV category was associated with future ADRD among males (3.21, 1.60–6.46), but not females (1.61, 0.80–3.23), though the risk difference between sexes did not reach statistical significance (P-value for sex interaction 0.18).

In further secondary analyses, we repeated all testing with the population narrowed only to individuals age ≥ 65 years at the time of first qualifying visit with qualifying EKGs (n = 4517). As was appreciated in the cohort with all adults, an association between BPV/HRV category during the clinical assessment period and incident ADRD during the outcome surveillance period was only appreciated among those with high systolic BPV and low HRV (2.27, 1.38–3.74) (Supplemental Table 6). As above, there were insufficient events among those age < 65 years with an EKG for analysis.

In sensitivity analyses, we examined the association of ADRD using alternative EHR derived BPV measures. All four evaluated metrics of systolic BPV demonstrated a similar significantly positive association with incident ADRD (SD 1.23, 1.12–1.34; CoV 1.23, 1.13–1.34; AVR 1.20, 1.09–1.31; MMD 1.24, 1.13–1.37) (Fig. 2A). These associations persisted following stratification by sex (Fig. 2B) and age (Fig. 2C). While no sex differences were appreciated (P-values for interaction all > 0.05), systolic BPV demonstrated a larger association with incident ADRD among those < 65 years of age when assessed by SD (P-value for age strata interaction 0.013), CoV (P-value for age strata interaction 0.002), and MMD (P-value for age strata interaction < 0.001), though not ARV (P-value for age strata interaction 0.06). Similar, though slightly lower, risk associations were appreciated with ADRD and all metrics of diastolic BPV (SD 1.16, 1.05–1.28, CoV 1.15, 1.04–1.26; AVR 1.23, 1.11–1.35; MMD 1.14, 1.08–1.20) (Fig. 2D), including following sex (Fig. 2E) and age (Fig. 2F) stratification.

In sensitivity analyzes using HRV derived from all qualifying EKGs, we found similar results, without a significant association between HRV and incident ADRD alone (Supplemental Table 7) or in combination with systolic BPV (Supplemental Table 8).

## Discussion

In this analysis of nearly 50,000 patients receiving continuous care, we found that increased BPV derived from real-world, clinically generated data was associated with incident ADRD over the ensuing 3 years. Notwithstanding the variability of clinical data routinely collected in patient care settings, BPV was effective in identifying patients are increased risk of developing ADRD in the intermediate term. Further, the association between clinically derived BPV and incident ADRD was robust to BPV metric and held across sex and age strata. By comparison, HRV in the clinical setting was not informative on its own and did not substantively add information on top of BPV. Taken together, these results indicate that clinically derived BPV, without need for considering HRV, may represent a potentially clinically useful marker of ADRD risk for a broad patient population.

Numerous mechanistic underpinnings have been proposed as driving the association between BPV, HRV, and ADRD ^22,23^. One set of hypotheses focuses on the potential for neurovascular damage imposed by both BPV and HRV which may result in neurocognitive dysfunction. Specifically, reduced HRV and increased BPV may result in reduced cerebral perfusion, microvascular damage, and white matter lesions ^6,24–27^. Alternatively, others postulate an alternative directionality linking dementia and BPV and HRV. Specifically, ADRD often results in autonomic dysfunction, particularly reduced parasympathetic and increased sympathetic tone, with patients suffering from orthostasis, dry mouth, and constipation ^28^. This school of thought hypothesizes that this abnormal and variable autonomic tone results in high BPV and low HRV, rather than BPV and HRV resulting in neurovascular damage and subsequent cognitive dysfunction ^7,29–32^. In this theory, high BPV and low HRV may serve rather as autonomic nervous system markers for subclinical cognitive impairment which becomes clinically recognized as the disease state progresses.

The rapidly expanding incidence and prevalence of ADRD demands novel, tailored approaches to population level screening. Age is well recognized as the single largest risk factor for the development of ADRD ^33^, however, as the number of individuals over the age of 65 grows, expected to reach 80 million by 2040, the healthcare system is under-resourced to enable global ADRD screening ^34^. Thus, targeted screening approaches and tools re needed to facilitate early diagnosis and, in turn, benefit patients, caregivers, and society alike. For patients, early diagnosis may allow for recognition of potentially reversible causes of cognitive impairment or treatment with disease modifying therapies. Additionally, early diagnosis allows patients to participate in clinical trials for novel treatment approaches and provides ample time for patient education and goal-setting discussions. For caregivers, early diagnosis affords opportunities for caregiver education, care planning, and access to community-based resources. At a societal level, early identification and treatment may help to limit downstream costs of care and identify patients eligible for future clinical studies ^35,36^. Overcoming the resource-demand mismatch in screening for ADRD necessitates a readily available marker of increased risk, allowing providers to direct screening efforts to those most likely to benefit, akin to AAA screening only among those at highest risk (males, over age 65, with a smoking history) ^37^. Based on our results, BPV represents an accessible clinical measure that could well fill this gap.

At the outset, we examined both BPV and HRV in association with ADRD risk in the clinical setting based on their ability to represent pathophysiologically related vascular as well as autonomic dysfunction and the abundant prior evidence linking these measures to ADRD outcomes. Importantly, previous reports of BPV and HRV associations with ADRD were primarily derived from clinical trial and cohort studies in which these metrics were assessed and recorded under ideal research conditions ^1–3^. It is well recognized that standardized methods used in clinical trials are rarely adhered to in clinical practice, potentially limiting the signal to noise ratio of an association with ADRD in clinically generated data ^9–11,38–41^. Further, no study of which we are aware has attempted to maximize on BPV and HRV as dual measures of autonomic function in ADRD risk assessment.

Our results demonstrate the use of clinically generated data does indeed provide sufficient signal to noise to assess ADRD risk. In particular, we found that individuals in both the highest quartile of BPV and lowest quartile of HRV were at twice the risk of developing future ADRD compared with those in the lowest and highest BPV and HRV quartiles, respectively. Importantly, when examined separately, BPV alone provided a similar ADRD risk estimate as combined BPV/HRV measures. In fact, use of HRV from single or pooled EKGs did not demonstrate predictive capacity for intermediate term incident ADRD risk. There are several potential explanations for our findings, including the possibility that higher BPV reflects vascular dysregulation that contributes mechanistically to brain atrophy and dysfunction ^42–46^ whereas HRV is a downstream manifestation of dysautonomia that develops after ADRD has already advanced ^28,42,47–49^. Another factor possibly contributing to lack of an HRV association may have been reduced sample size, with an 85% absolute reduction in the number of patients with eligible BP measurements compared to those with eligible EKGs. From a pragmatic perspective, any requirement for EKG data would also limit scalability for developing useful screening tools. Thus, our results indicating that a simplified and more accessible method of ADRD risk screening based on BPV alone is promising for broad implementation in the clinical environment. Notably, the association between BPV was consistent across race and sex strata, without significant between-group differences observed. Importantly, results were less robust among individuals < 65 years of age, largely related to the relatively small number of events in this cohort. While future larger studies may clarify the association between clinically derived BPV and ADRD among younger patients, our results emphasis the strength of this association predominately among those 65 and older. These findings further underscore the potential of using clinically generated BPV data to risk stratify patients across large patient populations. Given increasing evidence emphasizing the role of mid-life compared to later-life BP levels, along with the possibility of a U-shaped relationship between mean BP and ADRD risk, our findings also contribute to the growing literature on the importance as well as complexity of variations in BP measures in relation to longer-term outcomes. In this context, our study results highlight the need to further understand the dynamic combinations of risk traits predisposing to ADRD over the life course—as part of efforts to advance precision medicine approaches to disease screening and management. Our results overall also suggest that additional, less computationally complex measures may be promising for further development of screening approaches.

There are several limitations of our study that merit consideration. First, this was a retrospective analysis of patients receiving continuous care at a single center. Thus, the extent to which our results may represent causal relationships or are generalizable to other populations remains unclear and warrants further investigation. Importantly, given that we focused our study on patients receiving continuous care, the implications of our findings may be limited across the continuum of patients with less regular access to or utilization of healthcare services. While causation cannot be extrapolated from the results, this was also not the purpose of the study. Specifically, given the clear need for a tailored approach to ADRD screening, we sought to evaluate 2 clinical markers, together and independently, for their association with future dementia diagnoses. The causative nature and directionality of the relationship between BPV and HRV with ADRD remains to be fully explored, with intriguing and pathologically plausible explanations in both directions. We look forward to future work that further delineates the mechanisms underlying this association, while recognizing the immediate potential use of the finding for risk stratification. Concurrently, while both BPV and HRV are recognized as markers of autonomic tone, the degree to which long-term BPV and short-term HRV assess the same underlying biologic mechanisms remains unclear. Additionally, we cannot account for external factors such as patient positioning, recent exertion, or incorrect BP cuff size which may contribute to BPV and HRV. These factors exist as part of routine clinical practice and the aim of this study was specifically evaluate if the association between BPV, HRV, and ADRD demonstrated in controlled clinical trials is maintained even when ‘noise’ from these external variables is present. Finally, we relied on administrative data for the identification of both comorbid conditions and ADRD outcomes. Fortunately, these codes have been validated previously ^50,51^; further, the ADRD is more frequently under coded, which would balance our findings to the null ^52^.

Our findings indicate that clinically generated and readily available BPV data, without the need for additional HRV data, represents a novel metric by which to assess individual patient-level risk of future ADRD. This widely accessible and feasible use of EHR data offers a potential mechanism by which providers may efficiently identify patients most likely to benefit from comprehensive ADRD screening, the need for which will continue to burgeon with continued aging of the population.

## Methods

### Cohort development

Using data from the electronic health record (EHR), we identified patients receiving consistent ambulatory care in our health system, a large academic medical center in Southern California, during a ‘clinical assessment period’ from 2013 through 2016. Consistent care was defined as having at least 1 ambulatory care visit each calendar year during the clinical assessment period in which BP was documented. Systolic blood pressures < 60 mmHg or > 250 mmHg and diastolic blood pressures < 20 mmHg and > 200 mmHg were considered spurious and excluded. For this cohort, we extracted self-reported age, sex, race/ethnicity, and smoking status at the time of first qualifying visit. We further used ICD-9 and ICD-10 codes to identify baseline comorbid conditions including diabetes mellitus, chronic kidney disease, coronary artery disease, cancer metastases, myocardial infarction, heart failure, stroke, and atrial fibrillation or flutter (Supplemental Table 9). Dyslipidemia was not assessed due to previously recognized limitations in the accuracy of administrative coding, even in combination with laboratory data, when using EHR data to identify presence of this condition ^53^. We also determined if patients were prescribed an antihypertensive medication at any time during the clinical assessment period. We excluded individuals less than 18 years of age or with a history of ADRD (based on ICD codes or prescription for a dementia medication) prior to or during the clinical assessment period.

From this cohort, we identified a cohort of patients with an EKG from which HRV could be determined, excluding EKGs with evidence of atrial fibrillation or flutter, premature atrial or ventricular contractions, atrial or ventricular pacing, or missing leads (Supplemental Fig. 1). HRV was determined from EKGs rather than visit to visit heart rates as the former, but not the latter, has been shown to be positively associated with dementia ^3,6^.

### Blood pressure variability

Among qualifying individuals, we extracted all systolic (SBP) and diastolic (DBP) BPs, measured in mmHg, from every outpatient visit during the clinical assessment period; if multiple BP readings were recorded for a single visit, the SBPs and DBPs for that visit were averaged. Based on previously published literature examining multiple methods for the evaluation of BPV (i.e. standard deviation, coefficient of variation, and mean real variability), we elected to use variability independent of the mean (VIM) as our primary measure of visit-to-visit BPV, as alternate measures have been shown to be highly correlated with the mean BP, thus limiting their ability to differentiate from effects of mean BP ^54,55^.

Systolic and diastolic VIM were calculated separately. As previously described ^56^, VIM is calculated first as the standard deviation of BP readings divided by the mean BP raised to the power of *x*, where *x* is obtained from fitting a nonlinear regression model among the entire sample where standard deviation = a*mean^*x*^. This quantity is then multiplied by the sample mean BP raised to the power of *x*. As such,

$$ VIM = \frac{{k\,x\,Standard\,Deviation \,\left( {SBP} \right)}}{{Mean\,(SBP)^{x} }} $$

where

$$ k = Mean\,\left( {Mean\,\left( {SBP} \right)} \right)^{x} $$

Since VIM is derived from the distribution of BP within the sample itself, the values of VIM in a given sample cannot be compared to the values from a population with a different distribution of BP values. In general, the value of the VIM is a considered a relative, rather than an absolute measure of BP variability given that it is calculated in reference to values derived from mean BP; a higher value of VIM represents greater variability of visit-to-visit BP readings ^55^.

For use in sensitivity analyses, we calculated other commonly utilized BPV metrics for each patient including standard deviation (SD), coefficient of variation (CoV), average real variability (ARV), and maximum to minimum difference (MMD) ^57^. Calculation methods for additional BPV metrics is located in Supplemental Table 10. Assessments of the distributions and intercorrelations between measures of BPV can be found in Supplemental Tables 11 and 12.

### Heart rate variability

Among individuals with a qualifying EKG, QRS complexes were identified using peak finding algorithms from scikit-learn, from which we calculated Q-Q intervals in milliseconds, from all qualifying EKGs ^58^. We then calculated the root mean square of successive differences (RMSSD) for each qualifying EKG ^59^. Our primary analysis examined HRV using the most recent EKG from the clinical assessment period only. In sensitivity analyses, RMSSD was calculated as the mean RMSSD across all qualifying EKGs for each patient.

### Outcomes

We defined an ‘outcomes surveillance period’ of 2017 through 2019 during which we assessed for the development of incident ADRD based on ICD-9 and ICD-10 codes or the prescription of dementia medication (Supplemental Table 9). Of the n = 443 patients who developed ADRD during the surveillance period, a relevant dementia ICD code was present for 127 (28.7%), new dementia medication for 354 (79.9%), and both a dementia ICD and medication were present for 38 (8.6%). We identified all-cause death using vital status documented in the EHR. All study protocols were approved by the Cedars-Sinai Institutional Review Board with requirement for individual informed consent waived. All research was performed in accordance with the relevant guidelines and regulations.

### Statistical analyses

We performed multivariable Cox proportional hazards regression to compute hazard ratios (HRs) examining the association between BPV (separately for SBP and DBP), HRV, and a combination of BPV and HRV during the clinical assessment period and the development of incident dementia during the outcome surveillance period. For the combined BPV and HRV measure, we categorized patients into one of four groups, similar to work done by others ^47,60–62^: low BPV, high HRV (reference group); high BPV, high HRV; low BPV, low HRV; and high BPV, low HRV. High BPV values were those with VIM in the 75th percentile or above for the sample, and low HRV values were those with RMSSD in the 25th percentile or below for the sample. Analyzes were run using systolic BPV and performed on the largest qualifying sample (full qualifying cohort for models of BPV alone and EKG cohort for models including HRV).

Patients were censored at time of last recorded outpatient visit during which BP was measured or at the end of the outcome surveillance period (December 31, 2019), whichever came first. All analyses adjusted for age, sex, race/ethnicity, and smoking status along with presence of diabetes mellitus, chronic kidney disease, coronary artery disease, myocardial infarction, heart failure, stroke, or atrial fibrillation or flutter; all analyses also adjusted for use of antihypertensive medications, the number of visits at which a BP was recorded, number of qualifying EKGs, heart rate, and mean SBP and DBP. In secondary analyses, we repeated the primary outcome analyses in subgroups stratified by age (less than or ≥ 65 years of age at the time of first qualifying visit) and sex.

In sensitivity analyses, we repeated the primary BPV outcome analyses using various measures of BPV (SD, CoV, ARV, and MMD). We similarly repeated the primary HRV analyzes using the average RMSSD across all EKGs for each patient during the clinical assessment period. We conducted all statistical analyses using R (v3.6.1) and considered statistical significance as a two-tailed P value < 0.05.

### Standard protocol approvals, registrations, and patient consents

Study procedures were reviewed and approved by the Cedars-Sinai institutional review board (Study 00000603), with a waiver of informed consent.

### Supplementary Information

Supplementary Information.

## Data Availability

Due to the sensitive nature of the data collected for this study, requests to access the dataset from qualified researchers trained in protocols on the protection of human subjects may be sent to Cedars-Sinai Medical Center at biodatacore@cshs.org. JE and SC had full access to all the data in the study and takes responsibility for the integrity of the data and the accuracy of the data analysis.
