# Supplementary material for: Blood pressure variability supersedes heart rate variability as a real-world measure of dementia risk
Source: Sci Rep. 2024 Jan 22;14:1838. doi: 10.1038/s41598-024-52406-8 (PMC10800333; doi:10.1038/s41598-024-52406-8)
Supplement: Supplementary file 1 — Supplementary Information. [file 41598_2024_52406_MOESM1_ESM.docx]

**Blood Pressure Variability Supersedes Heart Rate Variability as a Real-World Measure of Dementia Risk**

Supplemental Materials

Supplemental Figure 1 … pg. 2

Supplemental Table 1 … pg. 3

Supplemental Table 2 … pg. 4

Supplemental Table 3 … pg. 5

Supplemental Table 4 … pg. 6

Supplemental Figure 2 … pg. 7

Supplemental Table 5 … pg. 8

Supplemental Table 6 … pg. 9

Supplemental Table 7 … pg. 10

Supplemental Table 8 … pg. 11

Supplemental Table 9 … pg. 12

Supplemental Table 10 … pg. 13

Supplemental Table 11 … pg. 14

Supplemental Table 12 … pg. 15

**Correspondence.** Joseph E. Ebinger, MD, MS, Department of Cardiology, Smidt Heart Institute, Cedars-Sinai Medical Center, Los Angeles, CA, USA, Phone (310) 423-2726**,** Email joseph.ebinger@csmc.edu

**Supplemental Figure 1.** Cohort Development Flow Diagram

**Supplemental Table 1.** Frequency of blood pressure readings per patient.

| **Number of Blood Pressure Recordings** | **Number of Patients** |
| --- | --- |
| 4 | 1983 |
| 5 | 2760 |
| 6 | 3248 |
| 7 | 3378 |
| 8 | 3450 |
| 9 | 3292 |
| 10 | 2955 |
| 11 | 2749 |
| 12 | 2370 |
| 13 | 2251 |
| 14 | 1943 |
| ≥15 | 17829 |

**Supplemental Table 2.** Multivariable Cox proportional hazards regression of demographic and clinical characteristics on incident Alzheimer’s Disease and Related Dementia diagnoses. VIM; variability independent of the mean.

|  | **Systolic VIM Model** | | **Diastolic VIM Model** | |
| --- | --- | --- | --- | --- |
| **Variable** | **HR (95% CI)** | **P Value** | **HR (95% CI)** | **P Value** |
| **Systolic VIM** | **1.24 (1.14, 1.35)** | **<0.001** | - | - |
| **Diastolic VIM** | - | - | **1.15 (1.05, 1.27)** | **0.003** |
| **Age (per year)** | **1.10 (1.09, 1.12)** | **<0.001** | **1.11 (1.10, 1.12)** | **<0.001** |
| **Male** | 1.09 (0.90, 1.31) | 0.37 | 1.08 (0.90, 1.30) | 0.42 |
| **Race/Ethnicity** |  |  |  |  |
| **White** |  |  |  |  |
| **Asian** | **0.44 (0.27, 0.72)** | **0.001** | **0.45 (0.28, 0.74)** | **0.002** |
| **Hispanic/Latinx** | 0.98 (0.68, 1.41) | 0.91 | 0.97 (0.67, 1.41) | 0.89 |
| **Non-Hispanic Black** | 1.15 (0.88, 1.49) | 0.31 | 1.15 (0.89, 1.50) | 0.29 |
| **Other** | 1.50 (0.96, 2.36) | 0.076 | 1.49 (0.95, 2.34) | 0.083 |
| **Smoking status** |  |  |  |  |
| **Current** |  |  |  |  |
| **Former** | 0.98 (0.57, 1.69) | 0.94 | 0.98 (0.57, 1.69) | 0.94 |
| **Never** | 1.05 (0.61, 1.80) | 0.87 | 1.05 (0.61, 1.79) | 0.87 |
| **Number of Outpatient Visits** | **1.01 (1.00, 1.01)** | **<0.001** | **1.01 (1.00, 1.01)** | **<0.001** |
| **Mean Systolic Blood Pressure (per 1 mmHg)** | 1.00 (0.99, 1.01) | 0.59 | 0.99 (0.98, 1.00) | 0.15 |
| **Mean Diastolic Blood Pressure (per 1 mmHg)** | 1.00 (0.98, 1.01) | 0.61 | 1.00 (0.98, 1.01) | 0.76 |
| **Comorbidities** |  |  |  |  |
| **Diabetes Mellitus** | **1.67 (1.37, 2.03)** | **<0.001** | **1.67 (1.38, 2.04)** | **<0.001** |
| **Chronic Kidney Disease** | 1.11 (0.89, 1.40) | 0.35 | 1.13 (0.90, 1.41) | 0.3 |
| **Atrial fibrillation/Atrial Flutter** | 0.93 (0.74, 1.18) | 0.57 | 0.92 (0.73, 1.16) | 0.47 |
| **Coronary Artery Disease** | 0.95 (0.77, 1.17) | 0.63 | 0.96 (0.78, 1.18) | 0.68 |
| **Use of antihypertensive medications** | 1.03 (0.86, 1.23) | 0.78 | 1.04 (0.87, 1.25) | 0.67 |
| **Metastatic Malignancy** | 0.99 (0.14, 7.09) | 0.99 | 1.04 (0.14, 7.44) | 0.97 |
| **Myocardial Infarction** | **1.74 (1.15, 2.65)** | **0.009** | **1.66 (1.09, 2.52)** | **0.018** |
| **Heart Failure** | **1.42 (1.08, 1.85)** | **0.01** | **1.40 (1.07, 1.83)** | **0.012** |
| **Stroke** | 1.08 (0.87, 1.33) | 0.5 | 1.10 (0.89, 1.36) | 0.4 |

**Supplemental Table 3.** Association heart rate variability and either systolic or diastolic blood pressure variability with incident Alzheimer’s Disease and Related Dementias among patients with an EKG.

|  | **EKG Cohort (n=7270)** | |
| --- | --- | --- |
|  | *Crude HR (95% CI)* | *Adjusted HR (95% CI)^1^* |
|  |  |  |
| **Systolic VIM Model** |  |  |
| Systolic VIM | **1.56 (1.36, 1.79)** | **1.37 (1.16, 1.61)** |
| HRV | 1.00 (1.00, 1.00) | 1.00 (1.00, 1.00) |
|  |  |  |
| **Diastolic VIM Model** |  |  |
| Diastolic VIM | **1.27 (1.09, 1.47)** | 1.18 (0.99, 1.40) |
| HRV | 1.00 (1.00, 1.00) | 1.00 (1.00, 1.00) |
|  |  |  |
|  |  |  |
| Abbreviations: CI, confidence interval; HR, Hazard Ratio; HRV; heart rate variability; VIM, variability independent of the mean  ^1^Cox models adjusted for age, sex, race/ethnicity, number of visits, use of antihypertensive medications, smoking status, diabetes mellitus, chronic kidney disease, atrial fibrillation/atrial flutter, coronary artery disease, mean systolic and diastolic blood pressure, presence of any metastatic malignancy, number of EKGs, myocardial infarction, heart failure, stroke, and heart rate. Patients censored at last follow up visit or death prior to the end of the study period, whichever occurred later. Analyses exclude patients of unknown race due to model convergence. | | |

**Supplemental Table 4.** C-Statistics for primary multivariable Cox proportional hazards regression of demographic and clinical characteristics on incident Alzheimer’s Disease and Related Dementia diagnoses, overall and stratified by sex, age, comorbidity status, and race/ethnicity. BP, blood pressure; HRV, heart rate variability; VIM, variability independent of the mean.

|  | **Systolic VIM (BP Cohort)** | **Diastolic VIM (BP Cohort)** | **HRV (EKG Cohort)** |
| --- | --- | --- | --- |
| **Pooled Analysis** | 0.89 | 0.887 | 0.80 |
| **Sex Stratified** |  |  |  |
| **Female** | 0.907 | 0.904 | 0.831 |
| **Male** | 0.868 | 0.865 | 0.748 |
| **Age Stratified** |  |  |  |
| **<65 years** | 0.852 | 0.838 | 0.839 |
| **≥65 years** | 0.76 | 0.755 | 0.722 |
| **Comorbidity Stratified** |  |  |  |
| **0 Comorbidities** | 0.905 | 0.904 | 0.843 |
| **≥1 Comorbidity** | 0.802 | 0.798 | 0.746 |
| **Race/Ethnicity Stratified** |  |  |  |
| **Non-Hispanic White** | 0.874 | 0.871 | 0.784 |
| **All Others** | 0.915 | 0.914 | 0.838 |

**Supplemental Figure 2.** Kaplan-Meier Curve of disease-free survival from Alzheimer’s Disease and Related Dementias by variability of systolic blood pressure above or below the median of the study cohort. VIM; variability independent of the mean.

**
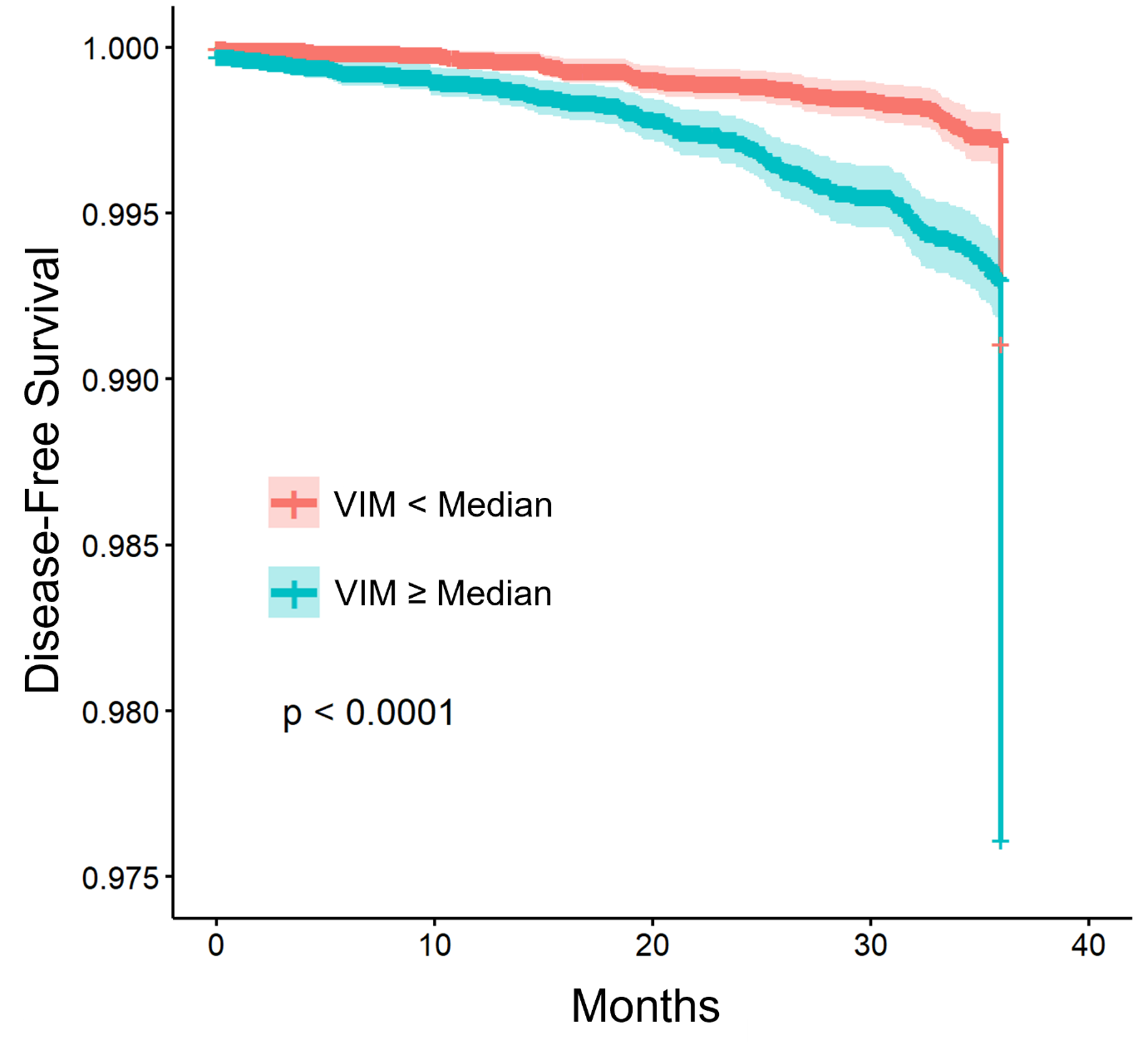
**

**Supplemental Table 5.** Association of quartiles of A) systolic blood pressure variability, diastolic blood pressure variability, and B) heart rate variability with incident Alzheimer’s Disease and Related Dementias.

1. Systolic and Diastolic Blood Pressure Variability. Analyses run on BP Cohort.

| **Outcome** | **BP Cohort (n= 48204)** |
| --- | --- |
|  | Adjusted HR 95% CI^1^ |
|  |  |
| **Systolic VIM** |  |
| Quartile 1 | **Ref** |
| Quartile 2 | 1.27 (0.90, 1.79) |
| Quartile 3 | **1.39 (1.01, 1.93)** |
| Quartile 4 | **1.85 (1.35, 2.53)** |
|  |  |
| **Diastolic VIM** |  |
| Quartile 1 | **Ref** |
| Quartile 2 | 1.32 (0.96, 1.80) |
| Quartile 3 | **1.38 (1.02, 1.87)** |
| Quartile 4 | **1.49 (1.10, 2.01)** |
|  |  |

| **Outcome** | **Overall (n= 48204)** |
| --- | --- |
|  | Adjusted HR 95% CI^1^ |
|  |  |
| **RMSSD** |  |
| Quartile 4 | **Ref** |
| Quartile 3 | 0.87 (0.54, 1.41) |
| Quartile 2 | 1.03 (0.66, 1.61) |
| Quartile 1 | 1.29 (0.81, 2.04) |
|  |  |

1. Heart Rate Variability. Analyses run on EKG Cohort.

Abbreviations RMSSD, root mean square of successive differences; VIM, variability independent of the mean

^1^Cox models adjusted for age, sex, race/ethnicity, number of visits, use of antihypertensive medications, smoking status, diabetes mellitus, chronic kidney disease, atrial fibrillation/atrial flutter, coronary artery disease, mean systolic and diastolic blood pressure, presence of any metastatic malignancy, number of EKGs, myocardial infarction, heart failure, stroke, and heart rate. Patients censored at last follow up visit or death prior to the end of the study period, whichever occurred later. Analyses exclude patients of unknown race due to model convergence.

**Supplemental Table 6.** Association of combined blood pressure and heart rate variability with incident Alzheimer’s Disease and Related Dementias in individuals aged 65 years and older the EKG cohort, overall and stratified by sex.

| **Outcome** | **Overall (n=4517)** | | **Female (n=2386)** | **Male (n=2131)** | **p-value^2^** |
| --- | --- | --- | --- | --- | --- |
|  | *Crude HR (95% CI)* | *Adjusted HR (95% CI)^1^* | *Adjusted HR (95% CI)^1^* | *Adjusted HR (95% CI)^1^* |  |
|  |  |  |  |  |  |
| **Systolic VIM + HRV** |  |  |  |  |  |
| Low VIM, High HRV (n=2401) | **Ref** | **Ref** | **Ref** | **Ref** | **-** |
| Low VIM, Low HRV (n=801) | 0.79 (0.46, 1.34) | 0.85 (0.49, 1.47) | 0.56 (0.23, 1.35) | 1.15 (0.57, 2.35) | 0.081 |
| High VIM, High HRV (n=937) | **1.64 (1.10, 2.43)** | 1.39 (0.93, 2.07) | 1.28 (0.75, 2.20) | 1.57 (0.86, 2.89) | 0.145 |
| High VIM, Low HRV (n=378) | **2.52 (1.57, 4.05)** | **2.27 (1.38, 3.74)** | 1.66 (0.82, 3.35) | **2.95 (1.43, 6.05)** | 0.912 |
|  |  |  |  |  |  |
| **Diastolic VIM + HRV** |  |  |  |  |  |
| Low VIM, High HRV (n=2481) | **Ref** | **Ref** | **Ref** | **Ref** | **-** |
| Low VIM, Low HRV (n=854) | 1.28 (0.84, 1.93) | 1.33 (0.86, 2.06) | 0.89 (0.46, 1.75) | **1.81 (1.01, 3.26)** | 0.161 |
| High VIM, High HRV (n=857) | 1.26 (0.83, 1.91) | 1.16 (0.76, 1.79) | 0.93 (0.52, 1.66) | 1.60 (0.85, 3.04) | 0.438 |
| High VIM, Low HRV (n=325) | 0.94 (0.45, 1.95) | 0.99 (0.47, 2.08) | 0.89 (0.34, 2.32) | 0.97 (0.29, 3.21) | 0.243 |
|  |  |  |  |  |  |
| Abbreviations: CI, confidence interval; HR, hazard ratio; HRV, heart rate variability; VIM: variation independent of the mean  1. Cox models adjusted for age, sex, race/ethnicity, number of visits, use of antihypertensive medications, smoking status, diabetes mellitus, chronic kidney disease, atrial fibrillation/atrial flutter, coronary artery disease, mean systolic and diastolic blood pressure, presence of any metastatic malignancy, number of EKGs, myocardial infarction, heart failure, stroke, and heart rate. Patients censored at last follow up visit or death prior to the end of the study period, whichever occurred later. Analyses exclude patients of unknown race due to model convergence.  2. P-values for sex interaction, i.e. difference in adjusted HRs between males and females. | | | | | |

*****There were too few events for analysis among individuals <65 years of age in the EKG Cohort (7 ADRD cases out of 2,753, 0.25%).

**Supplemental Table 7.** Association of heart rate variability with incident Alzheimer’s Disease and Related Dementias in the EKG cohort using all available EKGs.

| **Outcome** | **Overall (n=7270)** | | **Female (n=4015)** | **Male (n=3255)** | **p-value^2^** |
| --- | --- | --- | --- | --- | --- |
|  | *Crude HR (95% CI)* | *Adjusted HR (95% CI)^1^* | *Adjusted HR (95% CI)^1^* | *Adjusted HR (95% CI)^1^* |  |
|  |  |  |  |  |  |
| **Heart Rate Variability** | 1.00 (0.99, 1.00) | 0.99 (0.99, 1.00) | 1.00 (0.99, 1.00) | 1.00 (0.99, 1.00) | 0.194 |
| Abbreviations: CI, confidence interval; HRV, heart rate variability.  1. Cox models adjusted for age, sex, race/ethnicity, number of visits, use of antihypertensive medications, smoking status, mean systolic and diastolic blood pressure, diabetes, coronary artery disease, atrial fibrillation/atrial flutter, chronic kidney disease, amyloidosis, presence of any metastatic malignancy, heart rate, and number of EKGs. Patients censored at last follow up visit or death prior to the end of the study period, whichever occurred later.  2. P-values for sex interaction, i.e. difference in adjusted HRs between males and females. | | | | | |

**Supplemental Table 8.** Association of combined systolic blood pressure and heart rate variability with incident Alzheimer’s Disease and Related Dementias in the EKG cohort using all available EKGs, overall and stratified by sex.

| **Outcome** | **Overall (n=7270)** | | **Female (n=4015)** | **Male (n=3255)** | **p-value^2^** |
| --- | --- | --- | --- | --- | --- |
|  | *Crude HR (95% CI)* | *Adjusted HR (95% CI)^1^* | *Adjusted HR (95% CI)^1^* | *Adjusted HR (95% CI)^1^* |  |
|  |  |  |  |  |  |
| **Systolic VIM + HRV** |  |  |  |  |  |
| Low VIM, High HRV (n = 4149) | Reference | Reference | Reference | Reference | **-** |
| Low VIM, Low HRV (n = 1302) | 0.87 (0.52, 1.44) | 0.90 (0.53, 1.52) | 0.61 (0.26, 1.39) | 1.24 (0.62, 2.47) | 0.194 |
| High VIM, High HRV (n = 1303) | **2.03 (1.39, 2.98)** | 1.38 (0.93, 2.05) | 1.31 (0.77, 2.21) | 1.54 (0.84, 2.83) | 0.692 |
| High VIM, Low HRV (n = 516) | **2.62 (1.61, 4.27)** | **2.06 (1.23, 3.43)** | 1.22 (0.55, 2.71) | **3.08 (1.54, 6.16)** | 0.090 |
| Abbreviations: CI, confidence interval; HR, hazard ratio; HRV, heart rate variability; VIM: variation independent of the mean  1. Cox models adjusted for age, sex, race/ethnicity, number of visits, use of antihypertensive medications, smoking status, mean systolic and diastolic blood pressure, diabetes, coronary artery disease, atrial fibrillation/atrial flutter, chronic kidney disease, amyloidosis, presence of any metastatic malignancy, heart rate, and number of EKGs. Patients censored at last follow up visit or death prior to the end of the study period, whichever occurred later.  2. P-values for sex interaction, i.e. difference in adjusted HRs between males and females. | | | | | |

**Supplemental Table 9.** Administrative codes and medications for the identification of Alzheimer’s Disease and Related Dementias and pre-specified clinical comorbidities of interest.

|  | **ICD-9 and ICD-10 Codes** | **Medications** |
| --- | --- | --- |
| **Outcome** |  |  |
| Alzheimer’s Disease and Related Dementias | 46.1*, 291.2, 294.1*, 294.2*, 290.4*, 331.0-331.2, 331.4, 331.82, 332.0, 333.0, 797, A81.0*, F02.8*, F03.9*, F01.5*, F10.27, G31.0*, G31.1, G31.83, R41.81, G20, G23.1, G30.* | Aricept, Namzaric, Exelon, Razadyne, Reminyl, Cognex, Memantine |
| **Comorbid Conditions** |  |  |
| Coronary Artery Disease | 414.11, 414.12, 414.2, I25.1*, I25.7*, I25.8*, 411.1, 411.81, I25.4, I25.10, 414.0* |  |
| Diabetes Mellitus | 250.*, E10.*, E11.*, E13.*, E14.* |  |
| Atrial Fibrillation/Atrial Flutter | 427.31, 427.32, I48.0*, I48.1*, I48.2*, I48.3*, I48.4*, I48.9* |  |
| Hypertension | 403.00, 403.01, 403.10, 403.11, 403.90, 403.91, 404.*, 582.*, 585.*,586.*, 588.0, I12.0, I12.9, I13.0, I13.1, I13.10, I13.11, I13.2, I13.9, N25.0 Z49.0, Z49.1, Z49.2, Z94.0, Z99.2, 583.*, N03.*, N04.*, N05.*, N18*,N19* |  |
| Stroke | 362.3*, 430.*, 431.*, 433.*, 434.*, G46.*, I60.*, I61.*, I62.*, I63.*, I64.*, I65.*, I66.*, I67.*, S06.5 |  |
| Transient Ischemic Attack | 435.*, G45.0, G45.1, G45.8, G45.9, I67.848 |  |
| Concussion, Head Trauma, Traumatic Brain Injury | 959.01, 850.9, 854.0, S06.0X9A, S06.9*, S09.90 |  |
| Depression and Anxiety | 296.2*, 296.3*, 625.4, 293.83, 311, 648.4*, 293.89, 300*, F32.0-F32.5, F32.81, F32.9, F33*, F33.41, F33.42, N94.3, F06.30, O99.34*, F53*, F41.1-F41.9 |  |
| Metastatic Malignancy | 196.*-199.*, C77.*-C80.* |  |

**Supplemental Table 10.** Calculation methods for additional blood pressure variability metrics.

| **Blood Pressure Variability Metric** | **Calculation** |
| --- | --- |
| Standard Deviation | 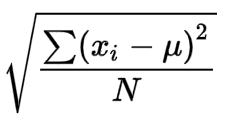Where,  N = size of population   - 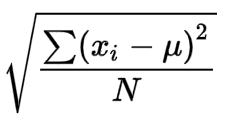= each BP value   = population mean |
| Coefficient of Variation | $\frac{Standard Deviation of BPs across all visits}{Mean BP across all visits}$ |
| Absolute Real Variability | $\frac{(\Delta\left( BP1 to BP2 \right)+\Delta\left( BP2 to BP3 \right)\ldots. + \Delta\left( BPn-1 to BPn \right))}{Total number of BP measures}$ |
| Maximum-Minimum Difference | Maximal BP value – Minimum BP Value |

Source: Yuichiro Yano, Visit-to-Visit Blood Pressure Variability—What is the current challenge?, American Journal of Hypertension, Volume 30, Issue 2, 1 February 2017, Pages 112–114, https://doi.org/10.1093/ajh/hpw124

**Supplemental Table 11.** Distribution of blood pressure variability measures calculated using various methods.

|  | **Mean (SD)** | **Median (IQR)** |
| --- | --- | --- |
| VIM Systolic | 10.87 (3.58) | 10.61 [8.48, 12.93] |
| VIM Diastolic | 7.59 (2.42) | 7.45 [5.99, 9.00] |
| SD Systolic | 10.94 (4.21) | 10.40 [8.08, 13.15] |
| SD Diastolic | 7.58 (2.44) | 7.43 [5.97, 8.99] |
| ARV Systolic | 11.84 (4.77) | 11.27 [8.77, 14.28] |
| ARV Diastolic | 8.00 (2.84) | 7.81 [6.30, 9.46] |
| MMD Systolic | 37.00 (17.53) | 34.00 [24.00, 46.00] |
| MMD Diastolic | 25.22 (10.44) | 24.00 [18.00, 31.00] |

VIM: variability independent of the mean; SD: standard deviation; ARV: average real variability; MMD: maximum-minimum difference

**Supplemental Table 12.** Intercorrelation of blood pressure variability measures calculated using various methods.

|  | **VIM Systolic** | **VIM Diastolic** | **SD Systolic** | **SD Diastolic** | **ARV Systolic** | **ARV Diastolic** | **MMD Systolic** | **MMD Diastolic** |
| --- | --- | --- | --- | --- | --- | --- | --- | --- |
| VIM Systolic | 1.000 |  |  |  |  |  |  |  |
| VIM Diastolic | 0.416 | 1.000 |  |  |  |  |  |  |
| SD Systolic | 0.838 | 0.463 | 1.000 |  |  |  |  |  |
| SD Diastolic | 0.395 | 0.991 | 0.480 | 1.000 |  |  |  |  |
| ARV Systolic | 0.526 | 0.350 | 0.721 | 0.367 | 1.000 |  |  |  |
| ARV Diastolic | 0.283 | 0.624 | 0.390 | 0.634 | 0.613 | 1.000 |  |  |
| MMD Systolic | 0.730 | 0.449 | 0.886 | 0.459 | 0.684 | 0.413 | 1.000 |  |
| MMD Diastolic | 0.397 | 0.836 | 0.491 | 0.840 | 0.422 | 0.603 | 0.626 | 1.000 |

VIM: variability independent of the mean; SD: standard deviation; ARV: average real variability; MMD: maximum-minimum difference.
